# Genetic Determinants of Primary Failure of Eruption: A Comprehensive Review of PTH1R Variants

**DOI:** 10.3390/genes17030279

**Published:** 2026-02-27

**Authors:** Benedetta Niccolini, Giulia Lauretti, Pietro Chiurazzi, Cristina Grippaudo, Elisabetta Tabolacci

**Affiliations:** 1 UOC Genetica Medica, Fondazione Policlinico Universitario A. Gemelli IRCCS, 00168 Rome, Italy; niccolini.bnd@gmail.com (B.N.); elisabetta.tabolacci@unicatt.it (E.T.); 2 Dipartimento di Scienze della Vita e Sanità Pubblica, Sezione di Medicina Genomica, Università Cattolica del Sacro Cuore, Largo F. Vito 1, 00168 Rome, Italy; 3 Neuroimmagini Lab, IRCCS Santa Lucia Foundation, Via Ardeatina, 309, 00179 Rome, Italy; 4 Dipartimento Universitario Testa Collo ed Organi di Senso, Università Cattolica del Sacro Cuore, 00168 Rome, Italy; 5 UOC Clinica Odontoiatrica, Dipartimento di Neuroscienze, Organi di Senso e Torace, Fondazione Policlinico Universitario A. Gemelli IRCCS, 00168 Rome, Italy

**Keywords:** primary failure of tooth eruption, *PTH1R* gene, tooth agenesis, dental anomalies

## Abstract

Primary Failure of Eruption (PFE) is a disorder characterized by aberrant tooth eruption, in which one or more teeth fail to follow the physiological eruptive pathway and remain partially or completely embedded within the bone or soft tissues. Although the etiopathogenesis of PFE is not yet fully elucidated, several contributing factors have been identified, including genetic alterations, hormonal disturbances, and systemic conditions. An expanding body of evidence points to the centrality of genetic determinants in the etiopathogenesis of PFE, supporting its occurrence in both syndromic contexts and non-syndromic presentations. Non-syndromic forms are closely related to heterozygous variants in the Parathyroid Hormone 1 Receptor (*PTH1R*) gene, located on chromosome 3p21, which encodes a receptor essential for the regulation of bone and dental growth and development. In most cases, pathogenic variants result in a non-functional receptor. To date, a substantial number 50 *PTH1R* variants have been documented in individuals exhibiting a phenotype consistent with PFE, underscoring the central involvement of this gene in the disorder’s molecular basis. Advances in understanding the genetic contribution to PFE emphasize the need for early diagnosis, as timely identification of the condition can prevent secondary dental complications and reduce reliance in adulthood on invasive orthodontic or surgical interventions, including extractions, orthognathic surgery, and implant-supported rehabilitation. This review aims to provide a comprehensive analysis of the spectrum of *PTH1R* variants implicated in PFE, examining genotype–phenotype correlations and their implications for diagnostic strategies and clinical management.

## 1. Introduction

Tooth emergence is a tightly regulated biological process essential for the establishment of normal occlusion and oral function [1]. Disruption of this process may lead to clinically significant consequences, including occlusal dysfunction, reduced masticatory efficiency, and an increased risk of dental and periodontal complications, with potential aesthetic implications [2]. Failure of tooth eruption is classified into two main forms, primary and secondary, based on distinct etiological mechanisms and diagnostic implications [3]. The secondary form is typically associated with identifiable local factors, most often mechanical barriers such as supernumerary teeth or odontomas that impede tooth movement toward the occlusal plane [4]. In contrast, eruption failure occurring in the absence of any physical obstruction, with a patent eruptive pathway, reflects an intrinsic alteration of the regulatory mechanisms governing tooth emergence [5]. This clinical and biological scenario defines Primary Failure of Eruption (PFE), a rare but clinically significant disorder of dental development first described by Proffit and Vig in 1981 [6]. PFE most commonly affects posterior teeth, involving one to four quadrants and resulting in a posterior open bite associated with deficient vertical development of the alveolar process. The distribution of the disease is typically unilateral and asymmetric; however, bilateral involvement and extension to one or more posterior quadrants have also been reported [2]. Although PFE primarily affects permanent teeth, clinical manifestations may also be observed in the primary dentition [7]. When permanent teeth are affected, eruption may be absent or may arrest prematurely despite their sub-gingival position; in such cases, involvement tends to extend to all molars distal to the most mesially affected tooth, suggesting posterior propagation of the eruptive defect along the dental arch. In the mixed dentition, the clinical presentation appears more variable: although primary teeth may be affected, permanent teeth within the same quadrant may either be involved or erupt normally, reflecting a different temporal expression of the disorder [8].

In PFE, affected teeth exhibit an abnormal or absent response to orthodontic forces, a feature that often becomes evident only during attempted orthodontic treatment. Although these teeth are not initially ankylosed, the application of orthodontic forces fails to induce physiological tooth movement and instead promotes the development of ankylosis. As a result, orthodontic extrusion is not only ineffective but may also be potentially harmful and should therefore be avoided [9]. These biomechanical characteristics have important implications for treatment planning and underscore the importance of an accurate diagnosis in order to avoid inappropriate therapeutic interventions and to optimize clinical management. To date, PFE remains a challenging condition to identify, as it is largely recognized through a process of exclusion requiring careful evaluation of all potential causative factors [9]; this difficulty is further compounded by the fact that its etiology has not yet been fully elucidated [10]. Nevertheless, a genetic alteration with variable penetrance and expressivity is currently considered the most plausible pathogenic mechanism, as supported by reports of familial aggregation. Further evidence for a genetic basis of eruption disorders is provided by the observation that abnormal tooth eruption occurs as part of the clinical spectrum of several human syndromes, as documented in the Online Mendelian Inheritance in Man (OMIM) database. In these syndromic conditions, failure of tooth eruption represents one component of a broader genetic phenotype and is commonly associated with additional oral and/or extraoral abnormalities. Specifically, eruption failure in syndromic contexts may coexist with oral anomalies such as hypoplastic amelogenesis imperfecta, relative microdontia, and intrapulpal calcifications, as well as with extraoral manifestations, including nephro-calcinosis in enamel-renal syndrome (*FAM20A*), ophthalmic abnormalities in GAPO syndrome (*ANTXR1*), oculodental syndrome (*FGFR3*), Nance–Horan syndrome (*NHS*), and skeletal abnormalities in osteoglophonic dysplasia (*FGFR1*). Collectively, these syndromic forms are genetically distinct. In contrast, non-syndromic PFE has been associated with variants in several candidate genes, including Transmembrane protein 119 (*TMEM119*) and lysine (K)-specific methyltransferase 2C (*KMT2C*) and periostin (*POSTN*) [11].

Nevertheless, accumulated evidence indicates that *PTH1R* represents the primary and most consistently implicated gene in the majority of reported non-syndromic PFE cases. *PTH1R* encodes the parathyroid hormone/parathyroid hormone-related peptide receptor, a G protein-coupled receptor that plays a central role in skeletal and dentoalveolar development through the regulation of bone remodeling and cellular responses to parathyroid hormone signaling. Beyond its involvement in PFE, *PTH1R* mutations are known to cause other skeletal disorders, such as chondrodysplasia, Eiken syndrome, and metaphyseal chondrodysplasia, underscoring the pleiotropic effects of this receptor. The causal association between *PTH1R* and PFE was first established by Decker et al. [12], who identified pathogenic *PTH1R* variants in affected individuals, thereby providing the first molecular evidence linking this gene to the disorder. Since this initial report, a growing number of clinically well-characterized cases have been associated with additional *PTH1R* variants, further strengthening and consolidating the central role of this gene in the pathogenesis of PFE. Notably, *PTH1R* variants can be transmitted through either autosomal dominant [7,13] or, less frequently, autosomal recessive inheritance [14]. Among the *PTH1R* variants reported so far, the majority are heterozygous (86%), and most have been associated with a PFE phenotype (73%) [15]. Collectively, these findings have established *PTH1R* as a key molecular target in PFE research, fostering the development of experimental models, including murine disease models and systems based on disease-specific induced pluripotent stem cells (iPSCs) [5]. Such experimental platforms have, in turn, enabled more detailed and mechanistic investigations into the biological pathways involved in tooth eruption and their disruption in PFE.

In light of the central role of *PTH1R* in tooth eruption and its consistent implication in PFE, genetic variation affecting this receptor represents a key determinant of the disorder.

Accordingly, this review first provides a comprehensive and mechanistic overview of *PTH1R* biology, with particular emphasis on the receptor’s structural architecture, the functional relevance of its individual domains, and the molecular mechanisms underlying its activation and downstream signaling within the principal pathways in which it operates. It then presents an updated and original compendium of *PTH1R* variants associated with PFE reported between 2010 and 2025, derived from the systematic integration of published literature and ClinVar data. Variants were curated and interpreted according to ACMG criteria and mapped at the protein domain level. This structured and integrative approach aims to enhance molecular diagnosis and refine the clinical interpretation of the disorder.

**Figure 1:**
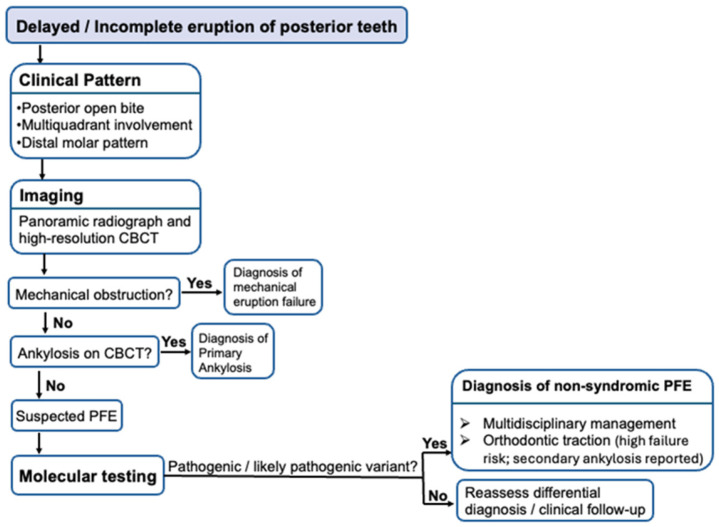
Diagnostic and management algorithm for suspected Primary Failure of Eruption (PFE). The flowchart outlines a structured clinical pathway beginning with delayed or incomplete eruption of posterior teeth and evaluation of characteristic features, including posterior open bite, multiquadrant involvement, and a distal molar pattern. Panoramic radiograph and high-resolution CBCT are used to exclude mechanical obstruction and primary ankylosis before establishing clinical suspicion of PFE. When clinical and radiological findings are consistent, molecular analysis of *PTH1R* and related genes may support the diagnosis. The algorithm highlights key management considerations, including multidisciplinary planning and the reported risk of orthodontic traction failure and secondary ankylosis in confirmed cases.

Given the diagnostic complexity of PFE and its significant therapeutic implications, we additionally propose a structured diagnostic and management flowchart designed to translate current evidence into practical clinical guidance, support differential diagnosis, and assist clinicians in identifying appropriate indications for genetic testing while avoiding inappropriate orthodontic interventions (Figure 1).

## 2. Molecular Structure and Signaling Properties of *PTH1R*

**Figure 2:**
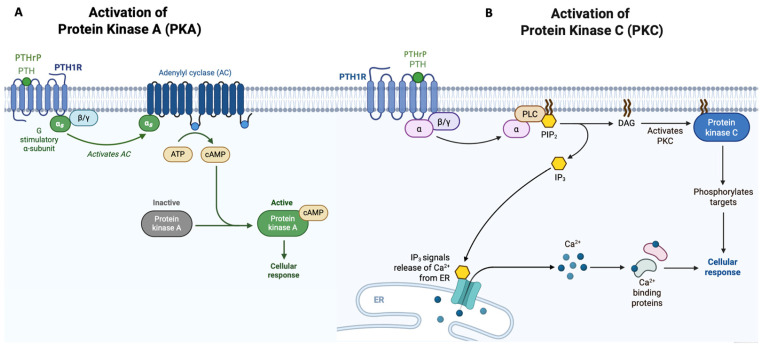
*PTH1R* signaling pathways. PTH/PTHrP binding to *PTH1R* induces activation of the cAMP/PKA pathway (**A**) and a PLC-dependent pathway leading to PKC activation (**B**).

At the molecular level, *PTH1R* is encoded by a genomic locus spanning approximately 26 kb and organized into 16 exons, 14 of which are protein-coding in the reference transcript (RefSeq: NM_000316.3). From a structural standpoint, *PTH1R* displays the typical architecture of this receptor class, comprising a large N-terminal extracellular domain (ECD), a transmembrane domain (TMD) composed of seven α-helices (I–VII), and an intracellular C-terminal domain. The extracellular domain, which encompasses approximately 150 amino acids, contains the principal ligand-binding site and mediates receptor–ligand interaction through a two-step binding mechanism. In this model, the C-terminal portion of the ligand initially engages the ECD, followed by interaction of the N-terminal portion with the TMD, a key event required for the conformational transition underlying receptor activation [16]. Complete removal of the C-terminal tail of *PTH1R* produces a receptor variant characterized by increased PTH-induced cAMP production [17]. This study, together with others, has shown that the C-terminal tail and the intracellular loops of *PTH1R* not only play a crucial role in receptor activation and deactivation, but also finely modulate receptor signaling through different subclasses of G proteins. Upon ligand binding, *PTH1R* can stabilize in distinct conformational states, including a G protein-coupled active state (RG) and an uncoupled state (R0). These states are associated with differences in duration and qualitative features of downstream signaling, contributing to the modulation of the cellular response [18]. Activation of *PTH1R* by its physiological ligands, PTH and PTHrP, promotes G protein coupling and triggers multiple intracellular signaling pathways through which the PTH/PTHrP axis regulates cellular growth, differentiation, and tissue remodeling [18]. The predominant signaling cascade is mediated by coupling to Gαs, leading to activation of adenylyl cyclase, increased intracellular levels of cyclic adenosine monophosphate (cAMP), and subsequent activation of protein kinase A (PKA) (Figure 2A). This pathway represents a central axis of *PTH1R*-mediated responses and supports transcriptional programs involved in the remodeling of mineralized tissues [19]. In parallel, *PTH1R* activates a PLC-dependent pathway, resulting in Ca^2+^ release and protein kinase C (PKC) activation (Figure 2B). This branch further broadens the biological output of *PTH1R* signaling by modulating cellular processes such as proliferation, differentiation, and local remodeling responses.

Within the bone compartment, particularly in osteocytes, *PTH1R* signaling has been linked to the regulation of key paracrine mediators involved in bone remodeling. In this context, the PTHrP–*PTH1R* axis modulates pathways including the SOST/WNT network and induction of RANKL, thereby contributing to the balance between osteoblastic and osteoclastic activity. Mechanistically, receptor activation leads to inhibition of SIK2, promoting nuclear translocation of HDAC4/5 and repression of MEF2C-dependent transcription, with consequent modulation of SOST expression. In parallel, dephosphorylation of CRTC2 promotes its nuclear translocation and activation of CREB, resulting in increased RANKL expression. Collectively, these mechanisms support the role of *PTH1R* as an integrative regulator of the molecular circuits underlying bone remodeling [20].

In the dentoalveolar compartment, where tooth eruption requires precise coordination between pericoronal bone resorption and basal bone formation, alterations in *PTH1R*-mediated signaling may disrupt local remodeling programs and contribute to the pathogenesis of primary failure of eruption (PFE). From this perspective, assessing the impact of *PTH1R* variants on receptor function and downstream signaling outputs represents a critical step toward establishing mechanistic links between molecular defects and the clinical phenotype.

### 2.1. PTH1R Expression

The expression pattern of *PTH1R* is consistent with the pleiotropic role of the PTH/PTHrP axis in skeletal development and bone remodeling [21]. Major sites of expression include bone, kidney, and cartilage, in line with the established role of this receptor in the regulation of skeletal physiology and mineral homeostasis. However, *PTH1R* expression is not restricted to these compartments, as the receptor has also been detected in extraskeletal tissues, including endothelium, liver, and smooth muscle [22]. Within the craniofacial district, *PTH1R* expression is particularly relevant to tooth development and eruptive processes. Specifically, the receptor is expressed in the dental follicle, the periodontal ligament (PDL), and the alveolar/orofacial bone, structures directly involved in the establishment of a dentoalveolar microenvironment permissive for normal eruption. In these compartments, PTHrP–*PTH1R* signaling has been associated with the regulation of cellular programs involved in periodontal tissue maturation and local remodeling.

### 2.2. Biological Role of PTH1R in Tooth Eruption and Implications for PFE

Tooth eruption is a complex and finely regulated biological process. Proper eruptive progression requires the normal development of the dental germ, composed of the enamel organ, the dental follicle, and the dental papilla, as well as a dynamic balance between bone formation at the base of the dental alveolus and resorption of the roots of deciduous teeth and the pericoronal bone [23]. This process is further supported by the proper functioning of the endocrine, bone metabolism, and neurogenic systems, whose integration is essential to ensure physiological eruption.

In this context, the parathyroid hormone receptor type 1 (*PTH1R*) plays a central role by regulating the activity of different cellular populations, particularly dental follicle cells, osteoblasts, and osteoclasts, as well as bone remodeling processes [24]. Through PTHrP-dependent signaling, *PTH1R* coordinates cellular programs within the dentoalveolar complex that are essential for establishing an eruption-permissive microenvironment. Mesenchymal stem cells of the dental follicle express high levels of PTHrP and the PTH1R receptor and are tightly regulated by the PTHrP–*PTH1R* signaling pathway [25]. Activation of *PTH1R* in mesenchymal progenitors of the dental follicle supports their proliferation and guides their differentiation, contributing to the formation of root cementum, the periodontal ligament (PDL), and alveolar bone, as well as promoting the coordinated signaling required to define the eruptive pathway. Experimental evidence from murine models demonstrates that conditional deletion of *Pth1r* in dental follicle progenitor cells results in severe eruption defects, including eruption failure, truncated root development, and abnormalities of cementum and the PDL, ultimately leading to ankylosis-like phenotypes. In particular, a study by Wanida et al. [26] showed that complete deletion of *Pth1r* in Osx^+^ dental follicle progenitor cells in mice (Osx-Cre; *Pth1r*^fl/fl^) leads to failure of tooth eruption, incomplete root development, tooth ankylosis, and absence of periodontal ligament insertion. In contrast, heterozygous deletion of *Pth1r* (Osx-Cre; *Pth1r*^fl/+) is associated with the formation of a thinner and structurally disorganized PDL. Additional evidence comes from a study by Takahashi [27] and colleagues, which demonstrated that dental follicle differentiation is altered in conditional *Pth1r* knockout mice (Pthrp-CreER; *Pth1r*^fl/fl^). In this model, follicle progenitor cells undergo premature differentiation into cementoblasts and immature osteoblasts, resulting in a weakly anchored periodontal ligament and failure of molar eruption, with a phenotype reminiscent of human PFE. In vitro studies have further clarified the role of the PTHrP–*PTH1R* signaling pathway in regulating dental follicle activity [28]. Reduced expression of chromodomain helicase DNA-binding protein 7 (CHD7) leads to a decrease in the osteogenic capacity of human dental follicle cells (hDFCs), whereas its overexpression promotes osteogenic differentiation. Moreover, increased *PTH1R* expression in CHD7-knockdown hDFCs is able to partially compensate for the impaired osteogenic differentiation, indicating that CHD7 modulates osteogenesis in hDFCs through regulation of *PTH1R*. In addition, cementoblasts have been shown to express *PTH1R* mRNA and to exhibit increased levels of cAMP and c-fos in response to PTHrP, suggesting a direct involvement of PTHrP–*PTH1R* signaling in cementogenesis [29].

Taken together, this evidence demonstrates that the PTHrP–*PTH1R* signaling system plays a key role in dental follicle differentiation and function, as well as in root development. Consequently, it is plausible to hypothesize that mutations in the *PTH1R* gene may disrupt normal follicular and root development, leading to the failure of tooth eruption characteristic of PFE. Beyond its role in the dental follicle, *PTH1R* also plays a crucial role in regulating alveolar bone remodeling, a process essential for eruptive movement [30]. Activation of *PTH1R* by endogenous PTH or PTHrP significantly influences bone physiology, exerting effects on multiple cell types, including osteocytes, osteoblasts, chondrocytes, and osteoclasts. By modulating molecular mediators that regulate osteoblastic and osteoclastic activity, the PTHrP–*PTH1R* axis ensures the balance between pericoronal bone resorption and basal bone formation required for tooth eruption. Dysfunction of *PTH1R*-mediated signaling may therefore impair local remodeling dynamics and prevent the coordinated bone turnover necessary for normal eruptive progression. Overall, these observations support a pathogenic model in which *PTH1R* dysfunction compromises both periodontal tissue maturation and alveolar bone remodeling, leading to failure of tooth eruption. This provides a strong biological rationale for the involvement of *PTH1R* in PFE and highlights the importance of characterizing *PTH1R* variants and their functional consequences to elucidate the molecular mechanisms underlying this condition.

### 2.3. PTH1R Variants Associated with the PFE Phenotype

Table 1 summarizes the spectrum of *PTH1R* variants identified through a comprehensive literature search conducted in PubMed and Google Scholar, including original articles and review papers published between January 2010 and December 2025. The search strategy specifically focused on studies reporting *PTH1R* variants associated with primary failure of eruption (PFE). Retrieved records were screened for relevance, and only variants explicitly reported in association with PFE were included. When the same variant was described in multiple publications, it was included only once in the final catalog, with all corresponding sources consolidated, in order to avoid duplication and redundancy. Table 2 summarizes *PTH1R* variants identified through systematic interrogation of the publicly available ClinVar database. The *PTH1R* gene name was used as the primary search term within the ClinVar interface. Variants classified as pathogenic, likely pathogenic, or variants of uncertain significance (VUS) were selected. Each variant was subsequently cross-checked using the Franklin platform to verify classification consistency and confirm its reported association with PFE. Variants associated exclusively with phenotypes other than PFE were excluded.

For both tables, inclusion was restricted to variants classified as pathogenic, likely pathogenic, or VUS. VUS were retained for completeness and transparency; however, their definitive contribution to the pathogenesis of PFE remains to be established. All variants were normalized using the reference transcript NM_000316.3 and described according to HGVS nomenclature.

Overall, a total of 77 PTH1R variants have been identified in association with PFE. From a molecular perspective, the reported variants comprise 45 missense variants, 13 nonsense variants, 7 frameshift variants, 9 splice-altering variants, and 3 in-frame or synonymous variants, highlighting a marked heterogeneity of mutational mechanisms.

**Figure 3:**
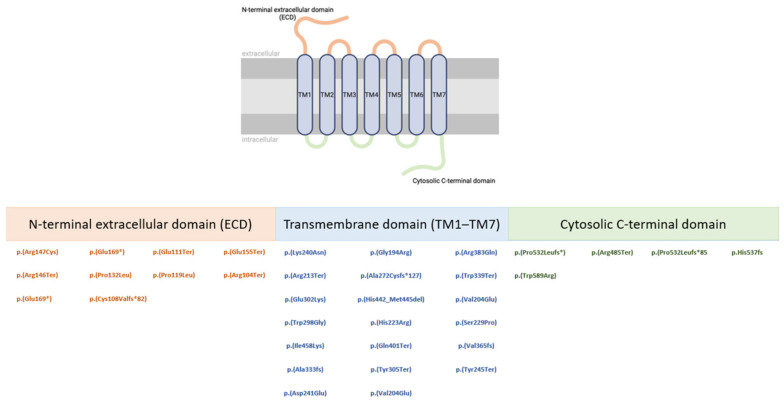
Structural organization of PTH1R and distribution of reported variants across receptor domains. The schematic illustrates the N-terminal extracellular domain (ECD), the seven transmembrane helices (TM1–TM7), and the cytosolic C-terminal domain of *PTH1R*. Only variants classified as pathogenic or likely pathogenic are shown and grouped according to their localization within each structural domain of the receptor.

The vast majority of variants in *PTH1R* are heterozygous de novo and familial missense variants. This is consistent with the autosomal dominant mechanism, in which a single altered allele is sufficient to disrupt normal receptor function (dominant negative effect). Missense variants are often localized in functionally important domains of the receptor, potentially affecting protein folding, ligand binding, or intracellular signaling. These alterations can impair the development and eruption of teeth, leading to the characteristic PFE phenotype. The presence of multiple missense variants in this gene in different individuals reflects both the existence of mutational hotspots and the variability of phenotypic expression. Segregation analysis within families can help identify the causative variant and support its classification as pathogenic or likely pathogenic, in accordance with ACMG guidelines. Pathogenic and likely pathogenic variants are further illustrated in Figure 3, which schematically depicts their distribution along the *PTH1R* protein sequence relative to its functional domains, highlighting the involvement of distinct structural regions of the receptor.

It should be noted that functional studies are lacking for many of these *PTH1R* missense and not variants, and their precise impact on receptor activity, signaling, and tooth eruption remains to be fully characterized.

A clear genotype-phenotype correlation between *PTH1R* variants and PFE has not been reported. They led to haploinsufficiency, presented variable penetrance and expressivity, even within the same family [11,32]. More “truncating” variants tend to produce a more severe phenotype (involvement of multiple dental quadrants) [32].

## 3. Conclusions

Primary eruption failure presents itself to clinicians as a difficult to identify condition due to the similarity of its clinical signs to ankylosis and mechanical eruption failure [41]. The signs associated with these conditions may overlap and vary in number and severity, complicating the differential diagnosis, particularly in cases of late presentation, familial cases or incomplete anamnestic information. In such situations, genetic testing of the *PTH1R* gene may represent a valuable adjunctive tool to support the diagnostic process and improve diagnostic accuracy. The clinical cases that are enriching the literature on the subject allow us to broaden the spectrum of *PTH1R* variants related to PFE. If the genetic test is positive, it is essential to classify the variants by type and pathogenicity. This can allow the dental clinician to look for associations between the presence of clinical signs and the variants presented by the patient. Diagnosis in childhood allows therapeutic errors that could have consequences on dental occlusion and jaw development to be avoided, and stimulates the search for new therapeutic solutions [41].

The future prospects are that genetic testing will become part of routine clinical practice, moving beyond the limited scope of research, to make diagnosis easier and more immediate.

Furthermore, the implementation of knowledge about the relationship between phenotype and genotype can help to better interpret the presence of clinical signs and guide the presumptive diagnosis, directing patients with a more obvious diagnostic doubt of PFE towards genetic testing.

## Figures and Tables

**Table 1:** List of *PTH1R* variant associated with PFE from the literature. Type of variant, its classification based on ACMG criteria and segregation and sources with PMID are reported. References are listed once per study and apply to the subsequent variants until a new reference is introduced.

Variant	Typology	ACMG Classification	Segregation	Ref.	PMID
c.1765T>C, p.(Trp589Arg)	Missense	PS4, PM2—Class 4 (LP)	familial	Grippaudo et al., 2018 [7]	29376733
c.64C>T, p.(Ala22Val)	Missense	PM2—Class 3 (VUS)	de novo	[7]	
c.1593delC, p.(Pro532Leufs *)	Frameshift deletion	PVS1, PM2—Class 4 (LP)	de novo	[7]	
c.611T>A, p.(Val204Glu)	Missense	PS2, PM2—Class 4 (LP)	familial	Jelani et al., 2016 [14]	n.a.
c.505G > T, (p.Glu169 *)	Nonsense	PVS1,PM2—Class 4 (LP)	familial	Grippaudo et al., 2019 [31]	31730001
c.1117-1G>A, p.(?)	acceptor splice site	PVS1, PS4, PM2—Class 5 (P)	de novo	Modafferi et al., 2024 [32]	39327493
c.720G>C, p.(Lys240Asn)	Missense	PS3, PM2—Class 4 (LP)	familial	[32]	
c.580G>C, p.(Gly194Arg)	Missense	PS4, PM2, PP3—Class 4 (LP)	familial	[32]	
c.988 + 5G>A, p.(?)	intron (splicing)	PM2, PP3—Class 3 (VUS)	familial	[32]	
c.356C>T, p.(Pro119Leu)	Missense	PS3, PS4, PM2—Class 5 (P)	familial	Izumida et al., 2020 [33]	31986066
c.463G>T p.(Glu155Ter)	Nonsense	PVS1, PM2, PP5—Class (P)	n.a.	Risom et al., 2013 [34]	n.a.
c.947C>A, p.(Ser316 *)	Nonsense	PVS1, PM2—Class 4 (LP)	n.a.	[34]	
c.989G>T, p.(Gly330Val)	Missense	PM2,PP3—Class 3 (VUS)	n.a.	[34]	
c.1082G>A, p.(Trp361 *)	Nonsense	PVS1, PM2—Class 4 (LP)	n.a.	[34]	
c.1348_1350del, p.(Phe450del)	in-frame deletion	PM2, PM4—Class 3 (VUS)	n.a.	[34]	
c.1050-3C>G, p.(?)	intron variant	PM2, PP3, PP5—Class 3 (VUS)	n.a.	[34]	
c.395C>T, p.(Pro132Leu)	Missense	PS3, PM2, PP3, PP5—Class 5 (P)	de novo	[34]	
c.892T>G, (p.Trp298Gly)	Missense	PM2, PP3, PP5—Class 4 (LP)	n.a.	[34]	
c.1148G>A, p.(Arg383Gln)	Missense	PP1, PS3, PM2—Class 4 (LP)	familial	Yamaguchi et al., 2011 [35]	21404329
c.322delT, p.(Cys108Valfs * 82)	Frameshift deletion	PVS1, PM2, PP3—Class 5 (P)	familial (heterozygous)	Roth et al., 2014 [36]	23771181
c.331G>T, p.(Glu111Ter)	Nonsense	PVS1, PM2—Class 4 (LP)	de novo	[36]	
c.436C>T, p.(Arg146Ter)	Nonsense	PVS1, PM2—Class 4 (LP)	n.a.	[36]	
c.543 + 1G>T, p.(?)	donor site loss	PVS1, PM2, PP3—Class 5 (P)	n.a.	[36]	
c.636dupT, p.(Arg213Ter)	Nonsense	PVS1, PM2—Class 4 (LP)	familial	[36]	
c. 639-2A>C, p.(?)	acceptor site loss	PVS1, PM2, PP3—Class 5 (P)	familial	[36]	
c.813dupT, p.(Ala272Cysfs * 127)	acceptor site loss	PVS1, PM2, PP3—Class 5 (P)	familial (heterozygous)	[36]	
c.1093delG, p.(Val365Cysfs * 141)	Frameshift	PVS1, PM2, PP5—Class 4 (LP)	familial (heterozygous)	[36]	
c.310C>T, p.(Arg104Ter)	Nonsense	PVS1, PS4, PM2—Class 5 (P)	familial (heterozygous)	[36]	
c.1324C>G, p.(His442Asp)	Missense	PM2, PP3—Class 3 (VUS)	de novo	[36]	
c.639-2A>G, p.(?)	splice-altering	PVS1, PM2, PP5—Class (P)	familial (heterozygous)	[36]	
c.1016G>A, p.(Trp339Ter)	Nonsense	PVS1, PM2—Class 4 (LP)	n.a.	[36]	
c.1036delC, p.(Leu346Trpfs * 9)	Frameshift deletion	PVS1, PM2, PP3—Class 4 (LP)	n.a.	[36]	
c.434A>G, p.(Tyr145Cys)	Missense	PM2, PP3—Class 3 (VUS)	n.a.	[36]	
c.590T>A, p.(Val197Glu)	Missense	PM2, PP3—Class 3 (VUS)	n.a.	[36]	
c.695T>G, p.(Leu232Arg)	Missense	PM2, PP3—Class 3 (VUS)	n.a.	[36]	
c.698G>A, p.(Arg233His)	Missense	PM2, PP3—Class 3 (VUS)	n.a.	[36]	
c.875T>C, p.(Leu292Pro)	Missense	PM2, PP3—Class 3 (VUS)	n.a.	[36]	
c.1142T>G, p.(Ile381Ser)	Missense	PM2, PP3—Class 3 (VUS)	familial	[36]	
c.1355G>A, p.(Gly452Glu)	Missense	PM2, PP3—Class 3 (VUS)	n.a.	[36]	
c.1736A>C, p.(Glu579Ala)	Missense	PM2, BP4—Class 3 (VUS)	familial	[36]	
c.1325_1336del, p.(His442_Met445del)	in frame deletion	PS4, PM2, PM4—Class 4 (LP)	familial	Zha et al., 2023 [37]	37480042
c.439C>T, p.(Arg147Cys)	Missense	PM2, PP3, PS2—Class 4 (LP)	de novo	[37]	
c.904G>A, p.(Glu302Lys)	Missense	PM1, PM2, PP2, PP3—Class 4 (LP)	familial	Lu et al., 2025 [38]	n.a.
c.1092delG, (p.Val365Cysfs * 141)	Frameshift	PVS1, PM2, PP5—Class 4 (LP)	n.a.	Rhoads et al., 2013 [39]	n.a.
c.543 + 1G>A, p.(?)	splice donor	PVS1, PM2, PP5—Class 4 (LP)	familial	Stellzig-Eisenhauer et al., 2010 [40]	20135246
c.463G>T, p.(Glu155 *)	Nonsense	PVS1, PM2, PP5—Class 5 (P)	familial	Grippaudo et al., 2021 [41]	34897565
c.1595delC, (p.Pro532Leufs * 85)	Frameshift	PVS1,PM2—Class 4 (LP)	n.a.	[41]	
c.64G>T, p.(Ala22Ser)	Missense	PM2—Class 3 (VUS)	n.a.	[41]	
c.337G>A, (p.Asp113Asn)	Missense	PM2, PP3—Class 3 (VUS)	n.a.	[41]	
c.354G>T, (p.Trp118CyS)	Missense	PM2, PP3—Class 3 (VUS)	n.a.	[41]	

n.a. not available.

**Table 2:** List of *PTH1R* variants associated with PFE from ClinVar database. Type of variant, its classification based on ACMG criteria and segregation.

Variant	Typology	ACMG Classification	Segregation	Refs.
c.668A>G, p.(His223Arg)	Missense	PS4, PM3, PM2, PP3—Class 4 (LP)	n.a.	CLINVAR
c.685T>C, p.(Ser229Pro)	Missense	PS4, PM2, PP3—Class 4 (LP)	n.a.	CLINVAR
c.1353 + 1G>T, p.(?)	splice donor	PS4, PVS1, PM2—Class 5 (P)	n.a.	CLINVAR
c.1201C>T, p.(Gln401Ter)	nonsense	PS4, PVS1, PM2—Class 5 (P)	n.a.	CLINVAR
c.996dup, p.(Ala333fs)	Frameshift	PVS1, PM2, PP5—Class (P)	n.a.	CLINVAR
c.915C>G, p.(Tyr305Ter)	Nonsense	PS4, PVS1, PM2—Class (P)	n.a.	CLINVAR
c.735C>G, p.(Tyr245Ter)	Nonsense	PVS1, PM2, PP5—Class (P)	n.a.	CLINVAR
c.75 + 1del, p.(?)	splice donor	PVS1, PM2, PP5—Class 4 (LP)	n.a.	CLINVAR
c.1603C>T, p.(Pro535Ser)	Missense	PM2—Class 3 (VUS)	n.a.	CLINVAR
c.1625C>A, p.(Thr542Asn)	Missense	PM2—Class 3 (VUS)	n.a.	CLINVAR
c.1621G>A, p.(Gly541Arg)	Missense	PM2—Class 3 (VUS)	n.a.	CLINVAR
c.1586A>G, p.(Asn529Ser)	Missense	PM2—Class 3 (VUS)	n.a.	CLINVAR
c.1574C>A, p.(Thr525Asn)	Missense	PM2—Class 3 (VUS)	n.a.	CLINVAR
c.1531C>G, p.(Arg511Gly)	Missense	PM2—Class 3 (VUS)	n.a.	CLINVAR
c.1481A>G, p.(Tyr494Cys)	Missense	PM2—Class 3 (VUS)	n.a.	CLINVAR
c.1462C>G, p.(Arg488Gly)	Missense	PM2—Class 3 (VUS)	n.a.	CLINVAR
c.1426C>T, p.(Arg476Cys)	Missense	PM2, PP3—Class 3 (VUS)	n.a.	CLINVAR
c.1420T>C, p.(Trp474Arg)	Missense	PM2—Class 3 (VUS)	n.a.	CLINVAR
c.1706C>T, p.(Ser569Leu)	Missense	PM2—Class 3 (VUS)	n.a.	CLINVAR
c.1696G>A, p.(Gly566Ser)	Missense	PM2—Class 3 (VUS)	n.a.	CLINVAR
c.1672C>T, p.(Pro558Ser)	Missense	PM2—Class 3 (VUS)	n.a.	CLINVAR
c.1665G>C, p.(Met555Ile)	Missense	PM2—Class 3 (VUS)	n.a.	CLINVAR
c.1756C>T, p.(Gln586Ter)	Missense	PM2, PVS1—Class 3 (VUS)	n.a.	CLINVAR
c.1753C>G, p.(Leu585Val)	Missense	PM2, PVS1—Class 3 (VUS)	n.a.	CLINVAR
c.1742C>G, p.(Pro581Arg)	Missense	PM2—Class 3 (VUS)	n.a.	CLINVAR
c.1739G>A, p.(Arg580Gln)	Missense	PM2—Class 3 (VUS)	n.a.	CLINVAR
c.1738C>T, p.(Arg580Trp)	Missense	PM2—Class 3 (VUS)	n.a.	CLINVAR

n.a. not available.

## Data Availability

The original contributions presented in this study are included in the article. Further inquiries can be directed to the corresponding author.
